# Supplementary material for: A comprehensive comparison of tools for fitting mutational signatures
Source: Nat Commun. 2024 Nov 2;15:9467. doi: 10.1038/s41467-024-53711-6 (PMC11530434; doi:10.1038/s41467-024-53711-6)
Supplement: Supplementary file 1 — Supplementary Information [file 41467_2024_53711_MOESM1_ESM.pdf]

# A comprehensive comparison of tools for fitting mutational signatures

## Supplementary Figures

Matúš Medo, Charlotte K.Y. Ng, Michaela Medová

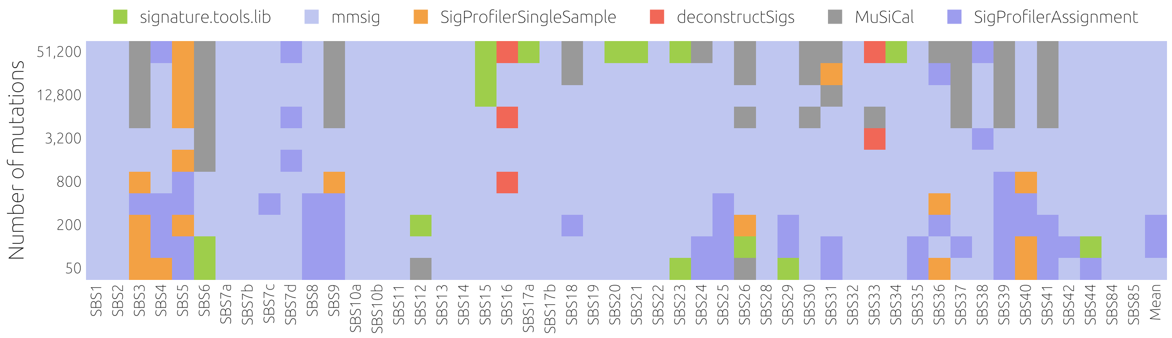


Supplementary Figure 1: Tools with the lowest average fitting error in single-signature cohorts for each signature and the number of mutations per sample (for each signature, we created one cohort with 100 samples).

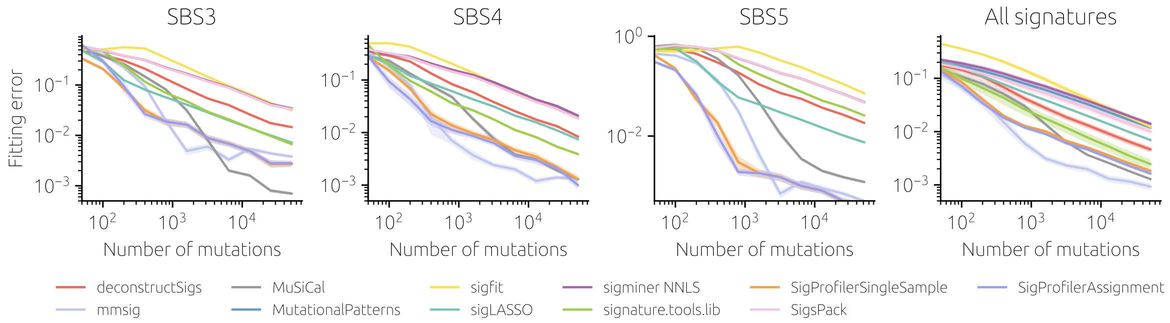


Supplementary Figure 2: As Figure 1c in the main text but with a logarithmic $y$-axis. A straight line with the slope of $-\beta$ in the log-log scale implies the power-law dependence $E \sim m^{-\beta}$ between the number of mutations per sample, $m$, and the fitting error, $E$. When the fitting error is averaged over all non-artifact signatures (last panel), linear fits to the empirical dependencies in the range $N \geq 1600$ yield exponents between 0.35 (for mmsig) and 0.59 (sigfit).

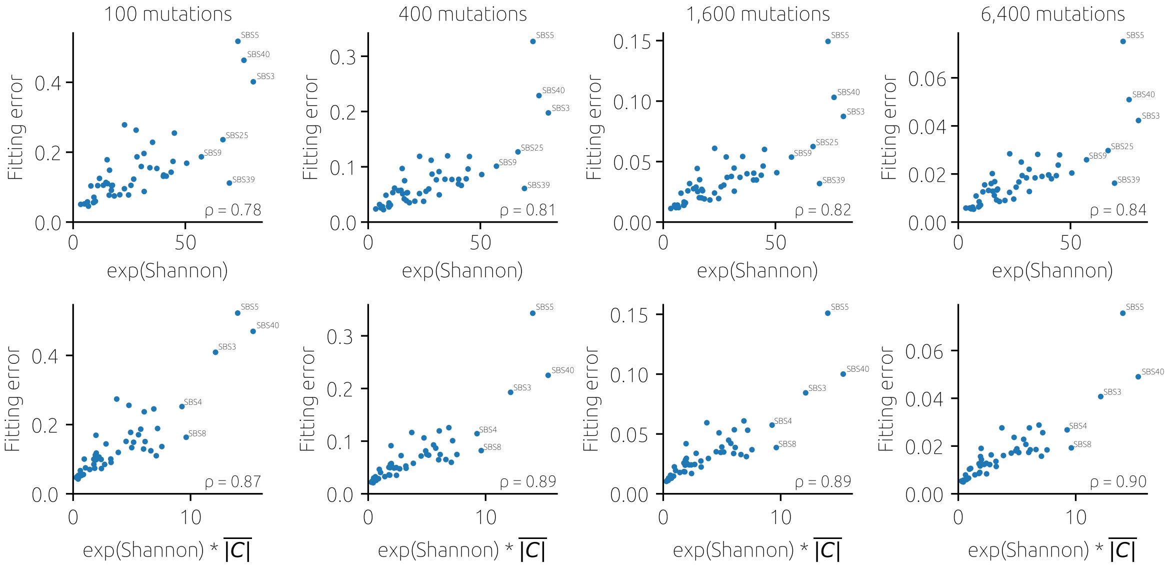


Supplementary Figure 3: Exponentiated Shannon entropy of signatures (top row) and exponentiated Shannon entropy multiplied with the signature's mean absolute Pearson correlation with all other signatures (bottom row) versus the average fitting error achieved by the evaluated tools (as in SF2, we exclude YAPSA, sigminer QP, and sigminer SA that produce similar results as MutationalPatterns). The exponentiated Shannon entropy (which measures an effective number of active nucleotide contexts in a signature) is closely related with the fitting error and the agreement improves with the number of mutations per sample. The agreement further improves when the average correlation with other signatures is taken into account. In summary, flat signatures that are on average similar to other signatures are the most difficult to fit.

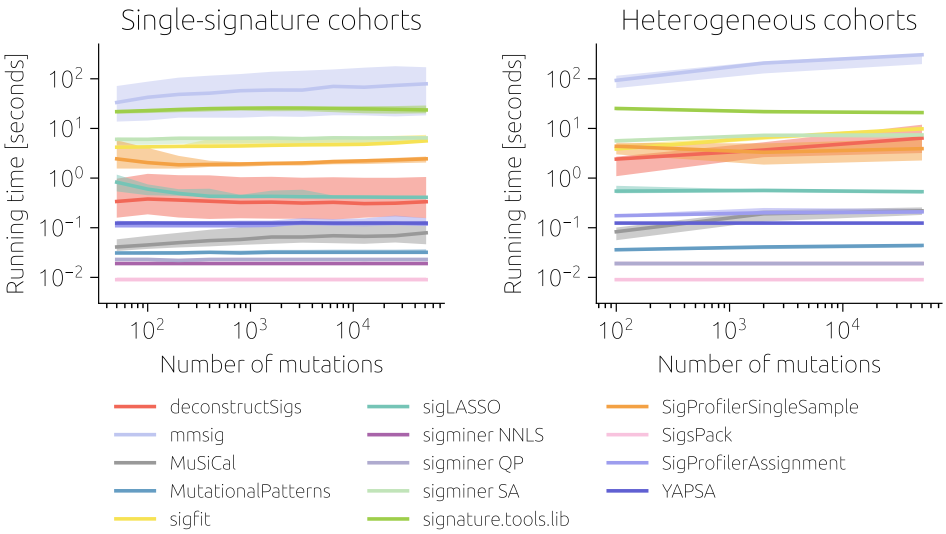


Supplementary Figure 4: The average running times (per sample) in single-signature cohorts (left) and heterogeneous cohorts (right) for all evaluated tools. In the right panel, sigminer QP and sigminer NNLS overlap. SigsPack and mmsig are the fastest and slowest tool in both cases; the ratios between their running times are 6,500 and 22,500 for single-signature and heterogeneous cohorts, respectively. deconstructSigs is more than 10 times slower in the heterogeneous cohorts. Simulations were run on Intel CPUs i5-6500 @ 3.20GHz.

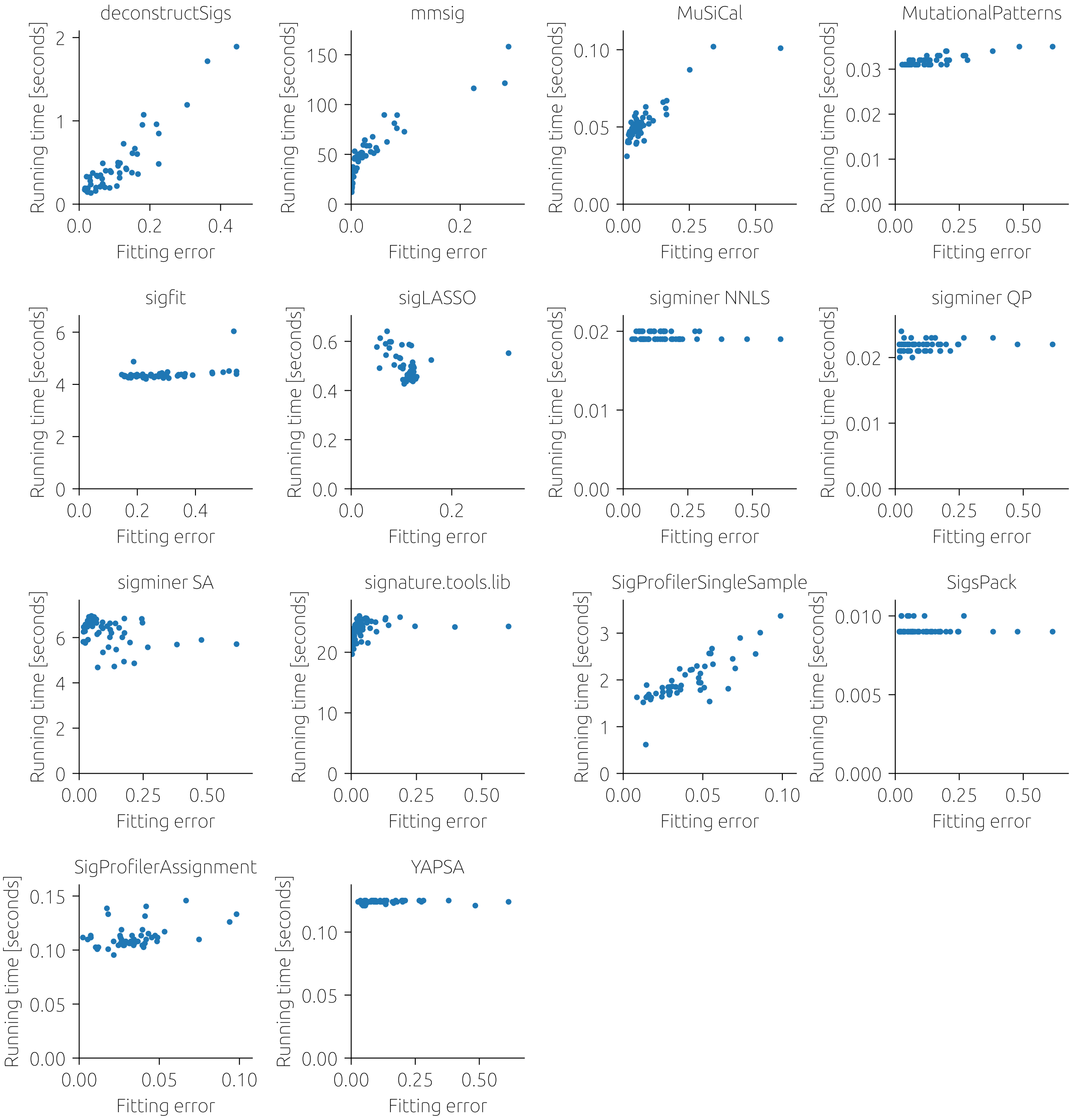


Supplementary Figure 5: The running times for single-signature cohorts plotted against the mean fitting error. The running times of three tools (deconstructSigs, mmsig, and SigProfilerSingleSample) grow with the signature difficulty.

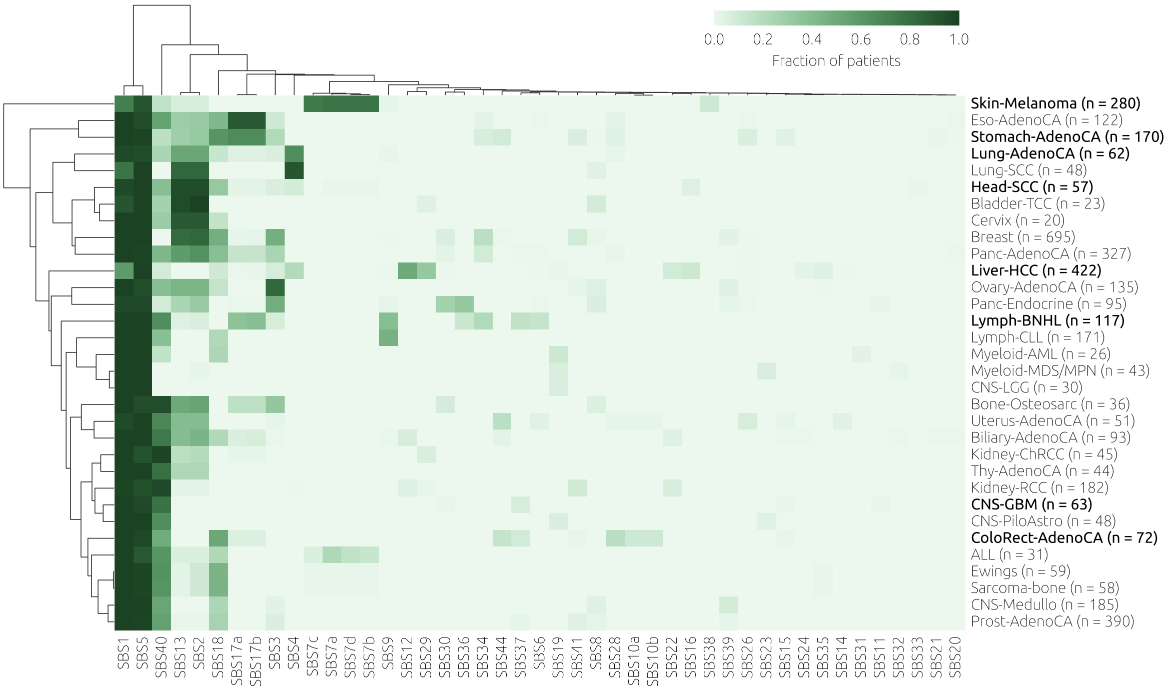


Supplementary Figure 6: The activity of signatures from the COSMICv3 reference catalog in the WGS-sequenced tissue data from various cancers at the COSMIC website (https://cancer.sanger.ac.uk/signatures/sbs/). The eight cancer types chosen for the evaluation of signature fitting tools are marked with bold.

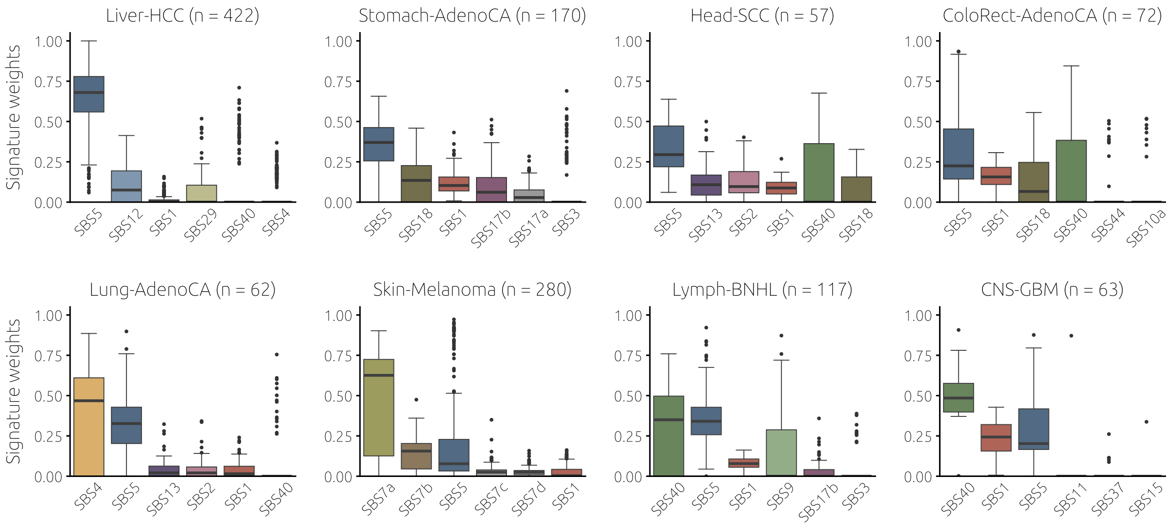


Supplementary Figure 7: Relative signature contributions in the heterogeneous cohorts. Empirical signature weights in WGS tissues from eight different cancer types (data obtained from the COSMIC website). Each panel shows six signatures with the highest median weight. The boxes show the quartiles of the data and the whiskers show the extend of the data up to 1.5-fold of the interquartile range; datapoints beyond the whiskers are shown as individual points (the same holds for all boxplots in our figures).

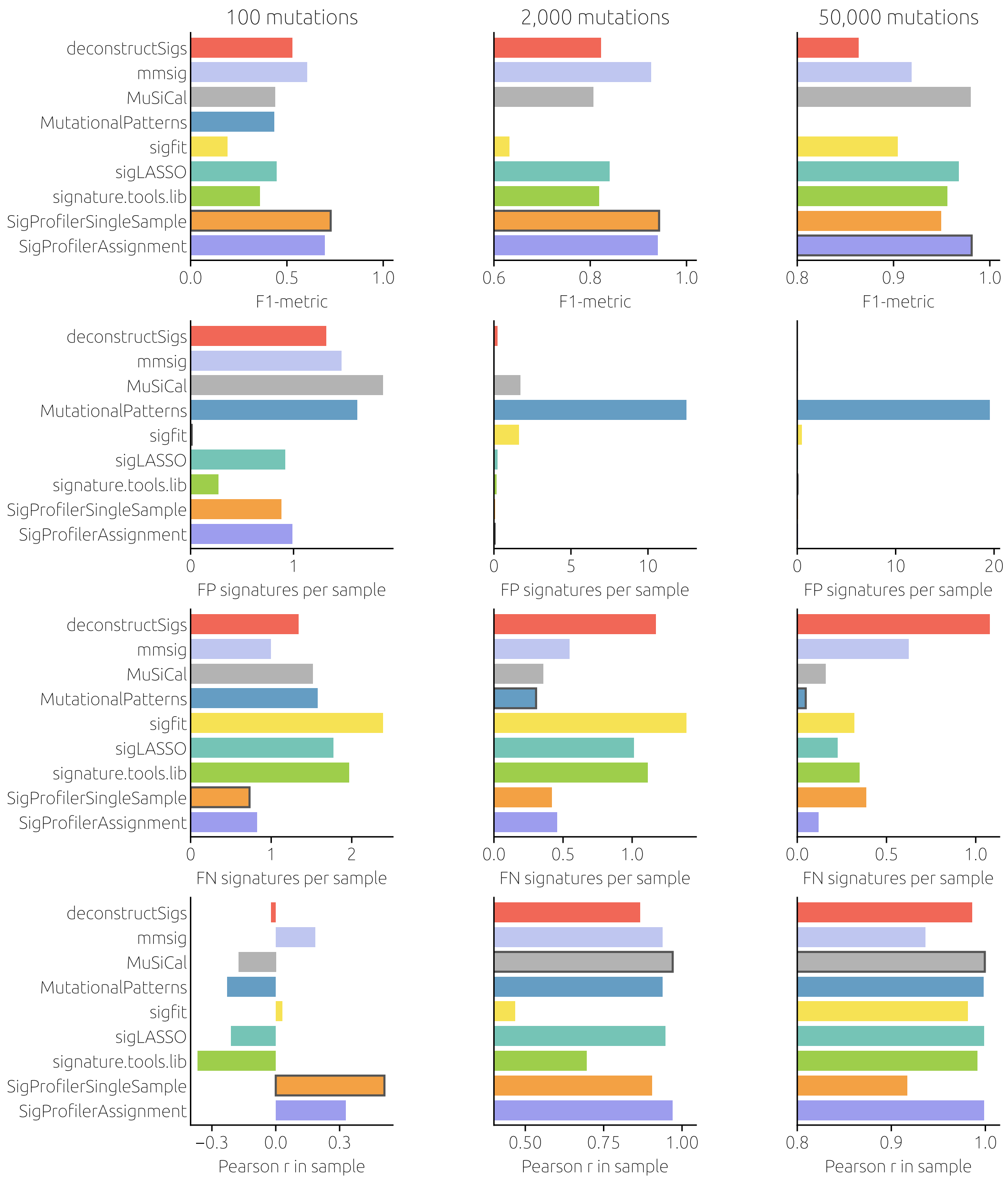


Supplementary Figure 8: Further evaluation metrics for the heterogeneous cohorts (a complement to Figure 2 in the main text).

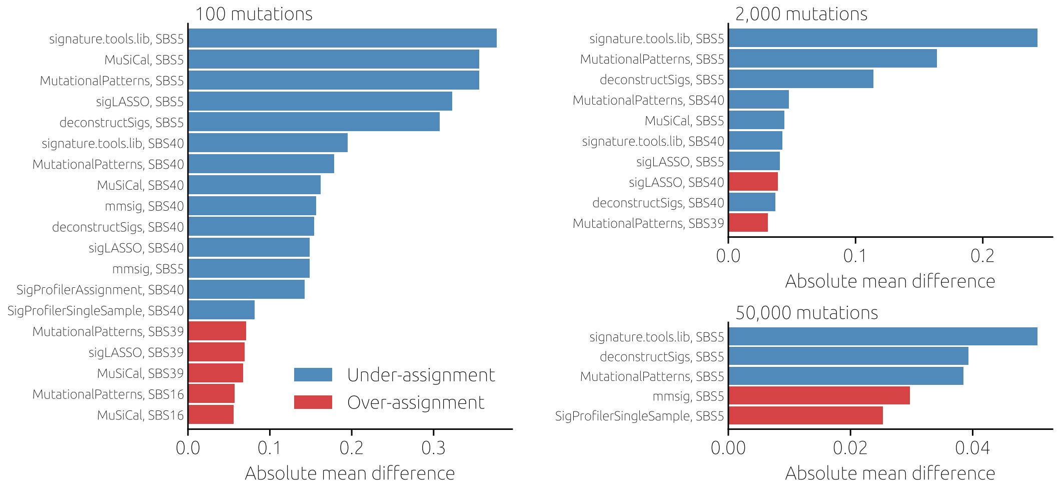


Supplementary Figure 9: Largest average differences between estimated and true signature weights for individual tools and signatures in the heterogeneous cohorts (a complement to Figure 2 in the main text).

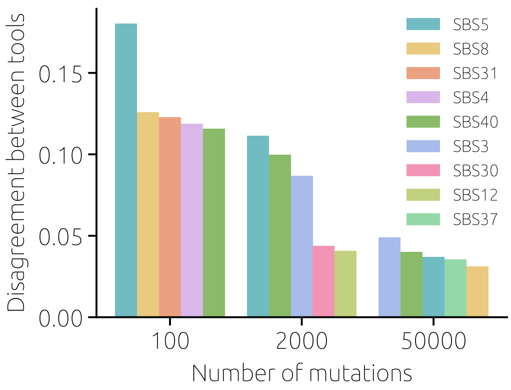


Supplementary Figure 10: Signatures with the largest disagreement between the results obtained by different tools in the heterogeneous cohorts (a complement to Figure 2 in the main text). The disagreement between tools is computed as the standard deviation of the weights estimated by different tools for a given sample, averaged over 50 cohorts with 100 samples for eight different cancer types.

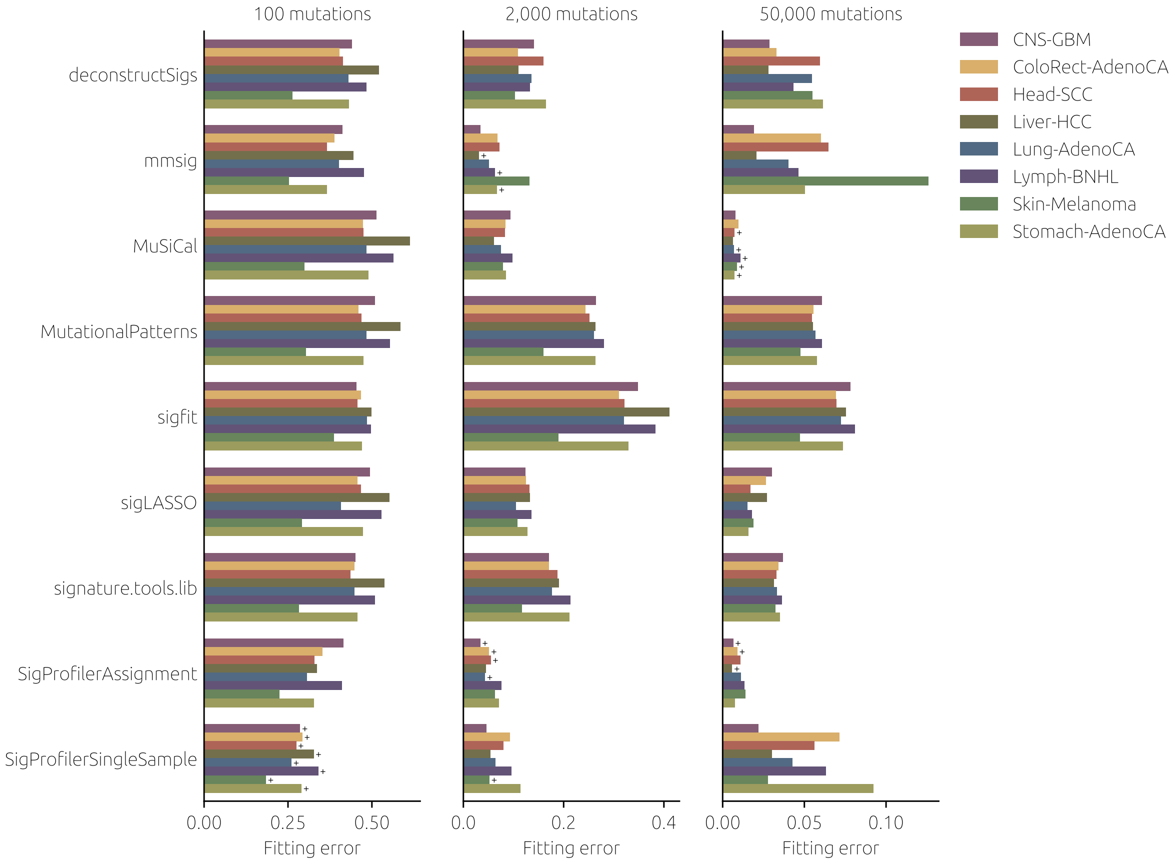


Supplementary Figure 11: Mean fitting error for the evaluated signature fitting tools stratified by the cancer type (a complement to Figure 2 in the main text). Plus symbols mark the best tool for each cancer type and a given number of mutations. The results are averaged over 50 cohorts with 100 samples for each number of mutations and cancer type.

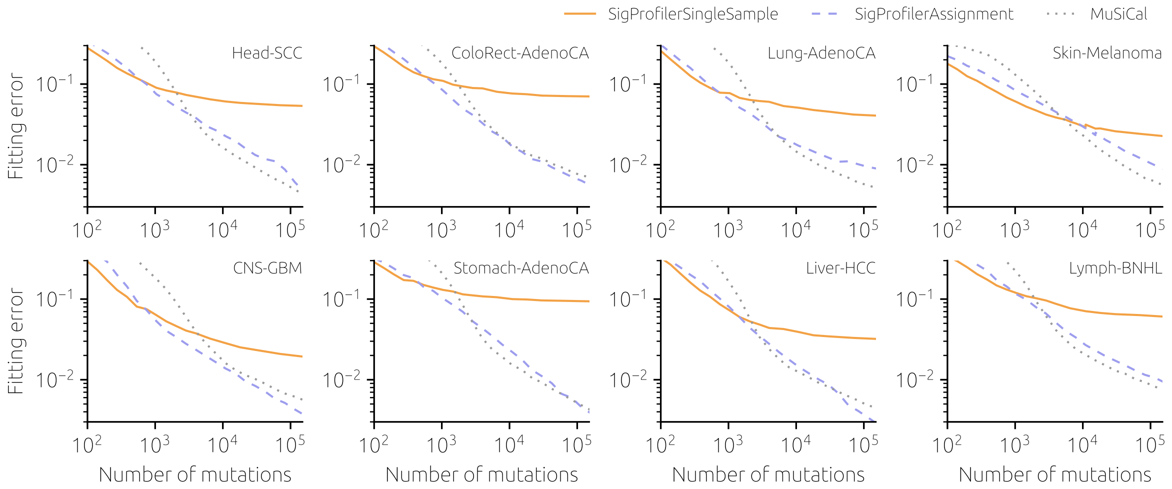


Supplementary Figure 12: The number of mutations for which SigProfilerSingleSample, SigProfilerAssignment, or MuSiCal achieve the lowest fitting error depends on the cancer type.

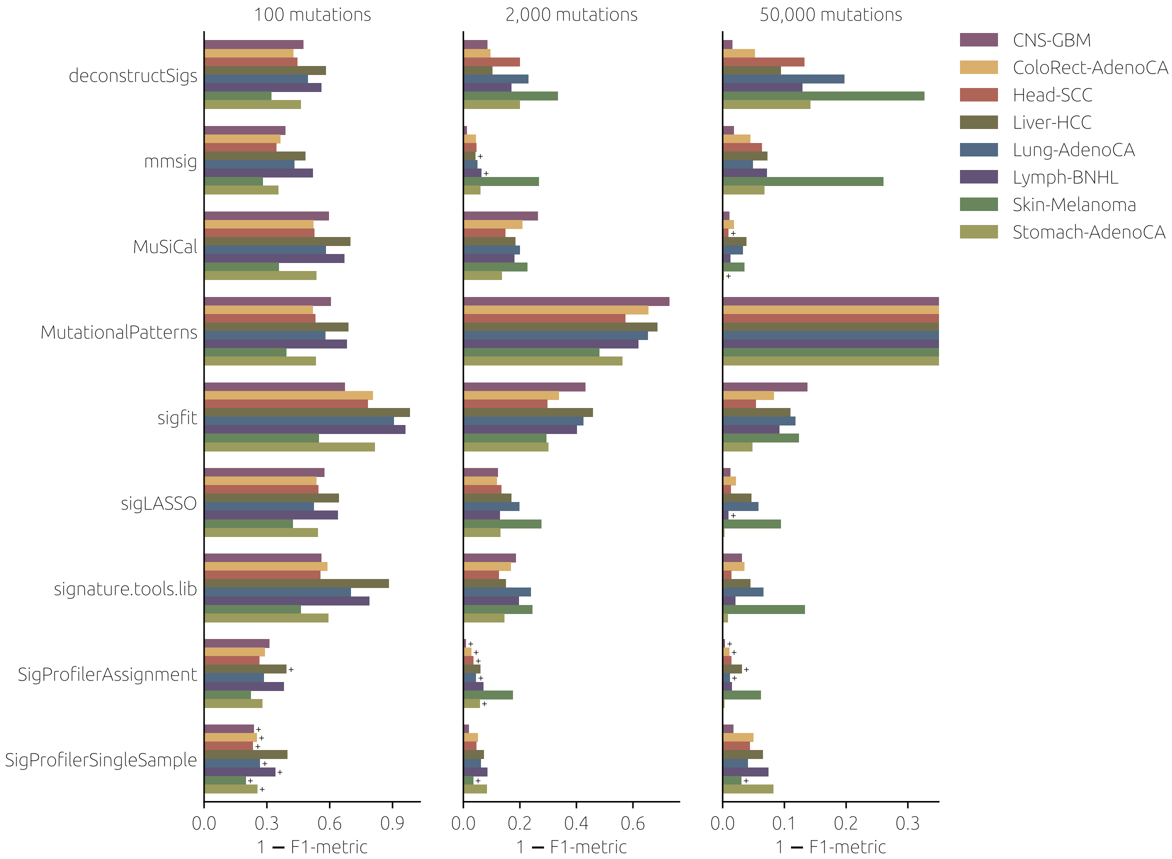


Supplementary Figure 13: As SF 11 but using a different evaluation metric, one minus the $F_1$ score (the lower the better). The ranking of tools is similar as in SF 11.

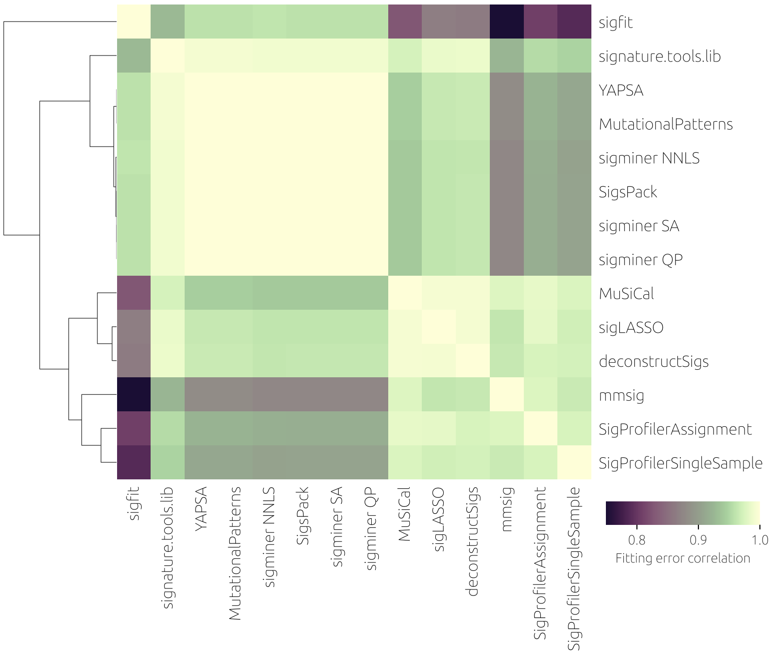


Supplementary Figure 14: Clustered heatmap of correlations between fitting errors achieved by the evaluated signature fitting tools on the heterogeneous cohorts.

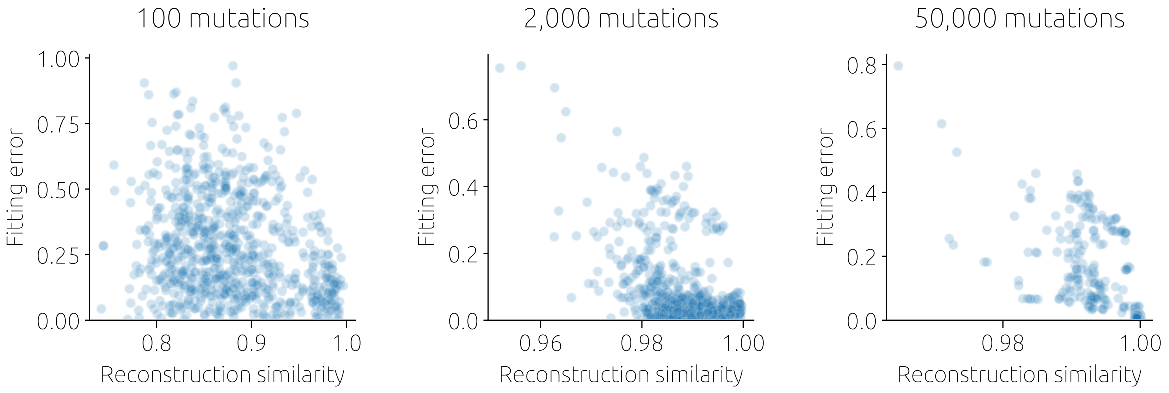


Supplementary Figure 15: Sample reconstruction similarity reported by SigProfilerSingleSample versus the sample fitting error in heterogeneous cohorts stratified by the number of mutations per sample. Note the different $x$-axis range between the panels which shows that no universal reconstruction similarity threshold can be chosen.

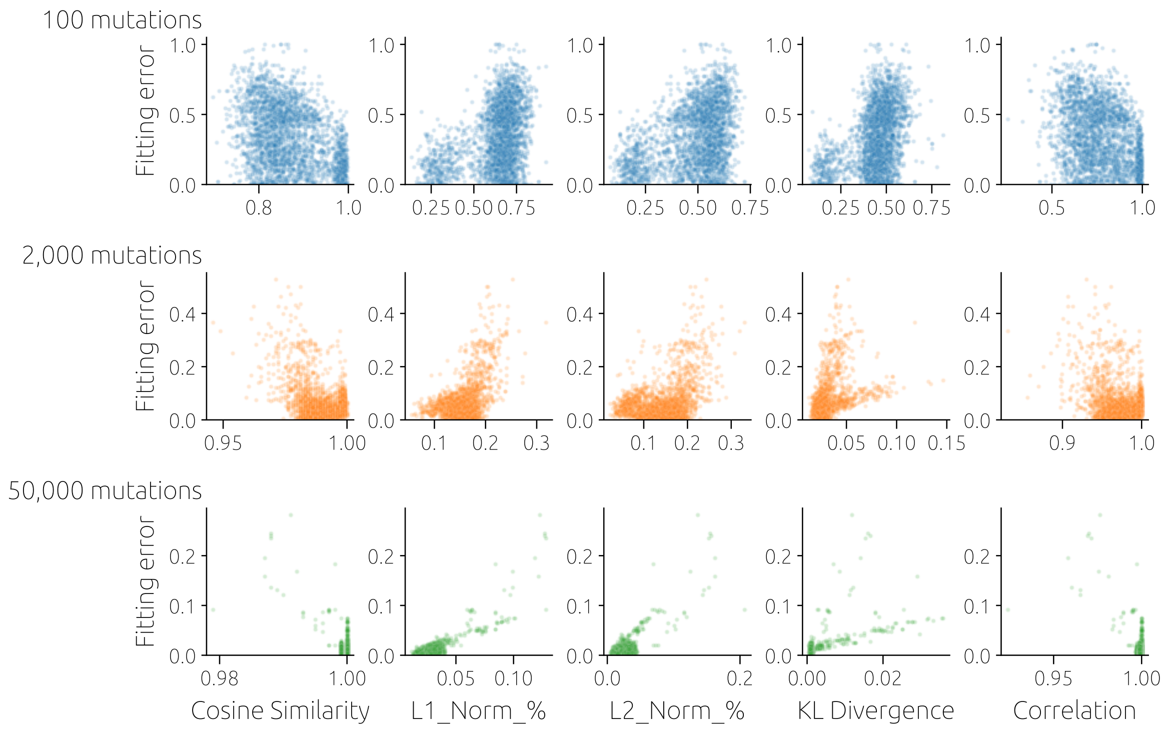


Supplementary Figure 16: Sample reconstruction quality metrics reported by SigProfilerAssignment versus the sample fitting error in heterogeneous cohorts stratified by the number of mutations per sample.

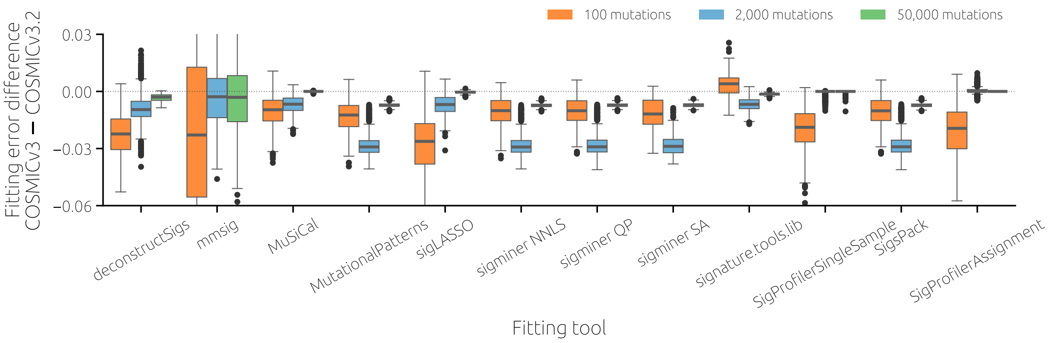


Supplementary Figure 17: Fitting error difference between the results obtained with COSMICv3 and COSMICv3.2, respectively, as a reference. Tool sigfit is omitted here because it failed to converge with COSMICv3.2. The differences are mostly negative as a result of the increased number of signatures included in COSMICv3.2 (78 as opposed to 67 for COSMICv3).

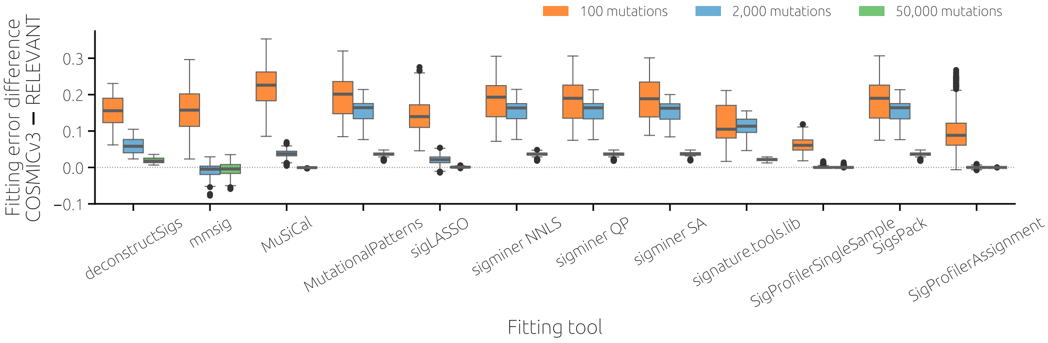


Supplementary Figure 18: Fitting error difference between the results obtained using COSMICv3 and only the relevant signatures (signatures that are active for a given cancer type and all artifact signatures), respectively, as a reference (50 cohorts with 100 samples for each of the eight cancer types). The differences are mostly positive as excluding the inactive signatures cannot induce any systematic errors in the estimated signature weights. For 2,000 and 50,000 mutations, SigProfilerAssignment does not benefit from using only the relevant signatures as a reference which means that it is able to automatically identify them even when complete COSMICv3 is used as a reference.

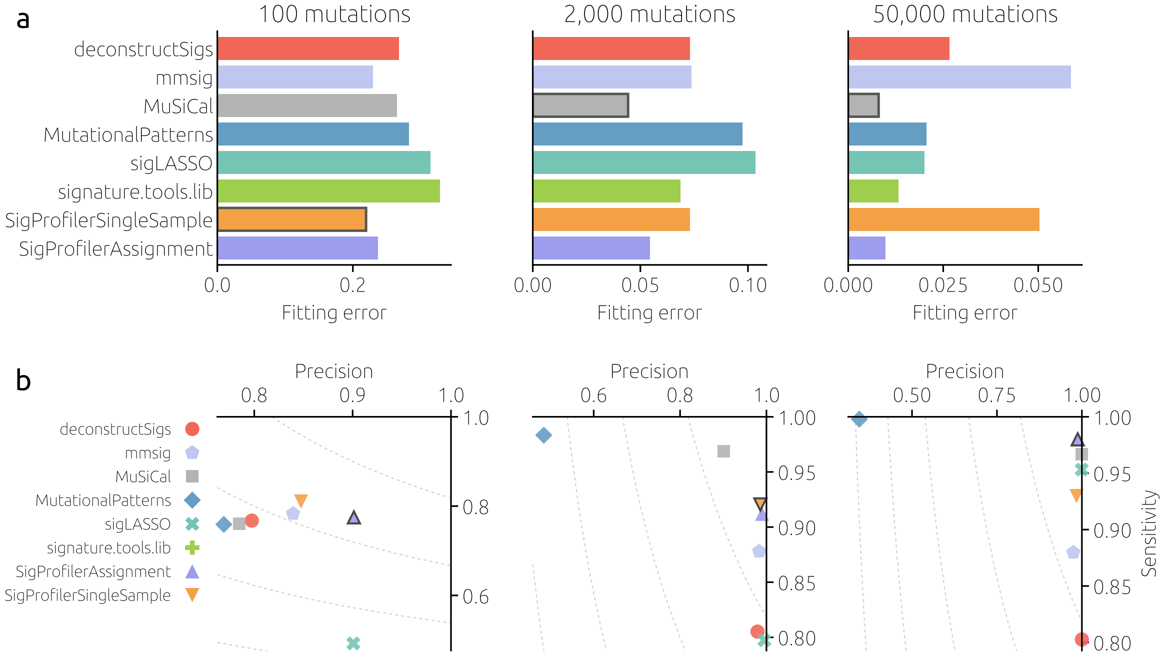


Supplementary Figure 19: A comparison of signature fitting tools for heterogeneous cohorts when only relevant signatures (*i.e.*, signatures previously identified in samples from a given cancer type) are used as a reference by the fitting tools. The results are averaged over 50 cohorts with 100 samples for each cancer type.

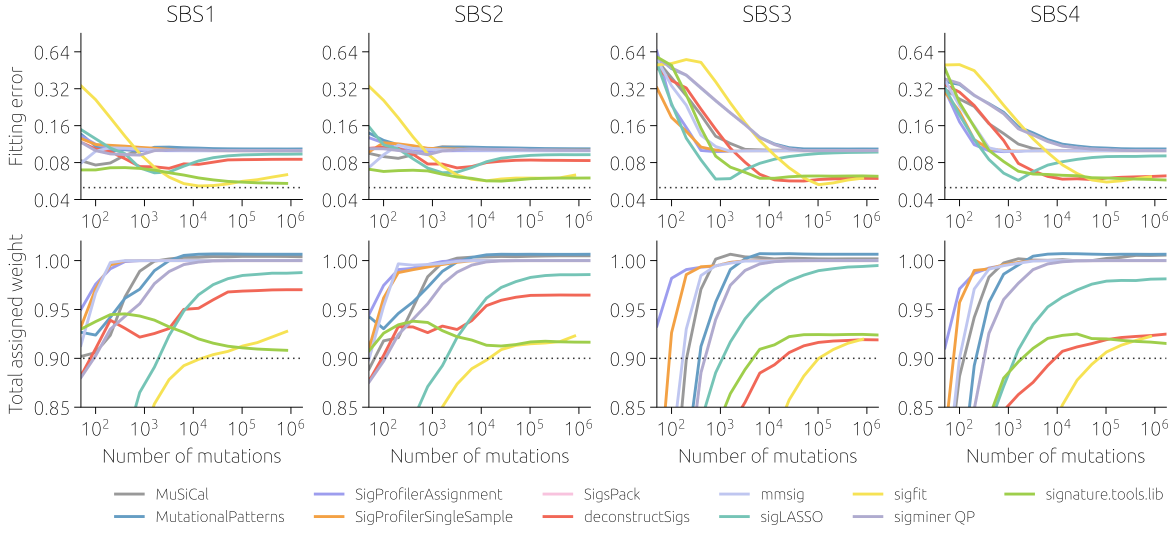


Supplementary Figure 20: A comparison of signature fitting tools for cohorts where 90% of mutations come from one signature (four columns corresponding to SBS1–4 being used as the main signature) and 10% of mutations come from a signature that is not in the reference catalog (SBS94 from COSMICv3.2 which is absent in COSMICv3 used by the evaluated tools as a reference). For each main signature and the number of mutations, the results are averaged over 100 samples in the generated cohort. Besides fitting error (top row), total relative weight assigned to the signatures (middle row), and the number of false positive signatures (bottom row) are shown. The dotted line shows the best possible performance of a fitting tool that does not have SBS94 in the reference catalog: Assigning the correct weight 0.9 to the main signature and 0 to all other signatures from COSMICv3 implies the fitting error of 0.05, total assigned weight 0.9, and zero false positive signatures. From all evaluated tools, signature.tools.lib comes closest to this best possible result. Two other tools that perform well here benefit from their conservative recommended practice. The authors of deconstructSigs recommend setting the relative weights below 0.06 to zero and the authors of sigfit recommend setting the relative weights whose estimated lower bounds of the 95% confidence intervals are below 0.01 to zero. This helps these two tools to differentiate from the other tools that assign all mutations to the reference signatures (this results in the fitting error of 0.1 and the total assigned weight is one).

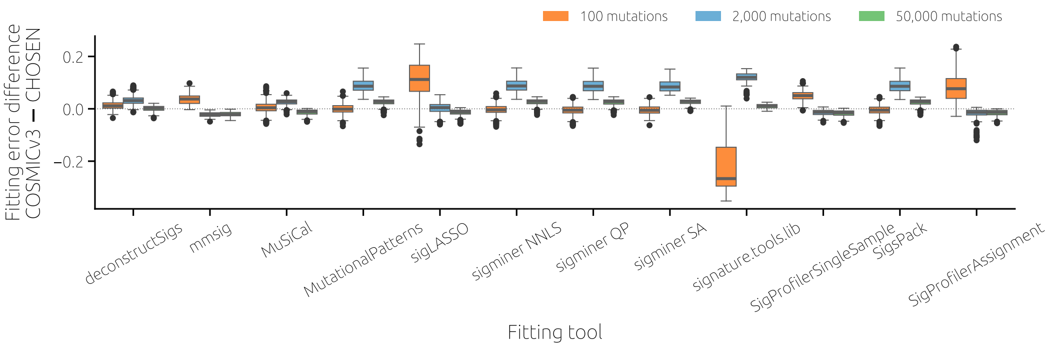


Supplementary Figure 21: Fitting error difference between the results obtained using COSMICv3 and a self-determined list of active signatures (two-step fitting process, see Methods in the main text), respectively, as a reference. A positive difference means that using a self-determined list of active signatures improves the results (*i.e.*, lowers the fitting error). For sophisticated and well-performing tools such as sigLASSO, mmsig, and SigProfilerAssignment, for example, the differences are positive only when the number of mutations per sample is small (100). By contrast, when the number of mutations per sample is high, the fitting error difference is mostly negative which shows that the process to determine the reference signatures is not beneficial.

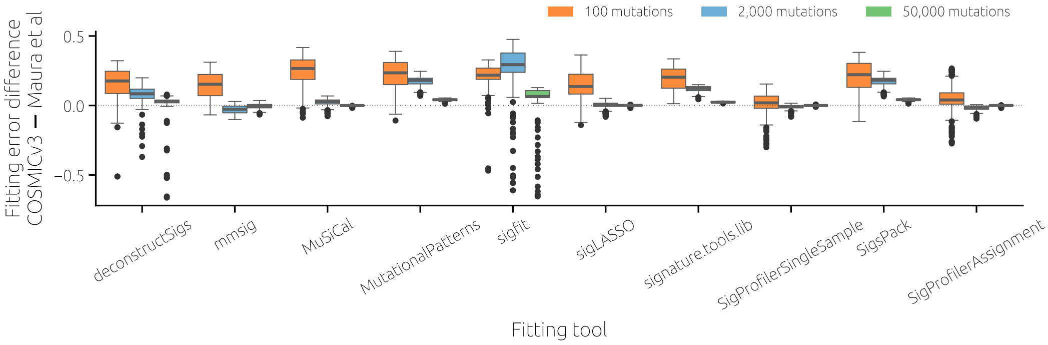


Supplementary Figure 22: Fitting error difference between the results obtained using COSMICv3 and the method by Maura et al. (see Methods in the main text), respectively, as a reference. A positive difference means that the method by Maura et al. improves the results (*i.e.*, it yields a lower fitting error).

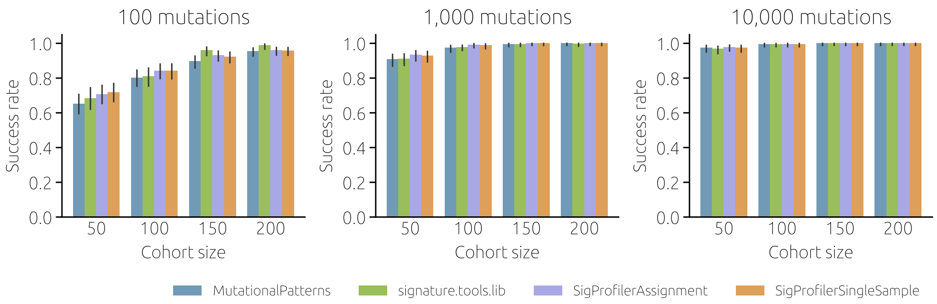


Supplementary Figure 23: As Figure 3b in the main text but systematic differences are introduced for signature SBS1 which is easier to fit than SBS40 used in Figure 3b. The success rate is defined as the fraction of 250 synthetic cohorts with artificially introduced differences in SBS1 weights between even- and odd-numbered samples where the estimated SBS1 weights differ significantly (Wilcoxon rank-sum test, $p$-value below 0.05). The errorbars show the 95% confidence interval (Wilson score interval). We see that for "easy" signatures, the differences between fitting tools tend to be smaller than for "difficult" signatures. SigProfilerAssignment and SigProfilerSingleSample maintain some limited advantage only for the smallest cohorts of samples with 100 mutations.

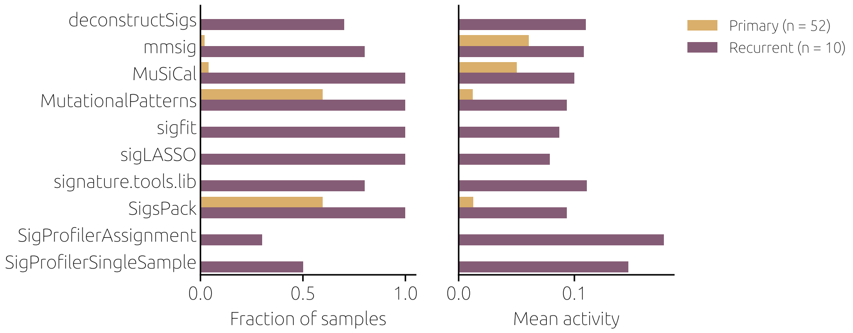


Supplementary Figure 24: Mutational signature analysis of untreated primary tumor ($n$ = 52) and treated recurrent tumor ($n$ = 10) samples from the OV-AU cohort for which SBS mutational catalogs are available. The recurrent samples have been treated with surgery and chemotherapy. As platinum-based anti-cancer drugs are the standard chemotherapy, the recurrent samples are presumed to be exposed to platinum. The fraction of samples where platinum signatures (SBS31 and SBS35) are active (left panel) and their mean joint activity in the samples where they are active (right panel). MutationalPatterns and SigsPack find a low activity of platinum signatures in 60% of the primary samples although these samples were not treated with platinum-based drugs. Most tools find platinum signatures in the majority of the recurrent samples. SigProfilerAssignment (30%) and SigProfilerSingleSample (50%) are more conservative in this respect and only attribute these signatures to the samples where their activity is high (right panel).

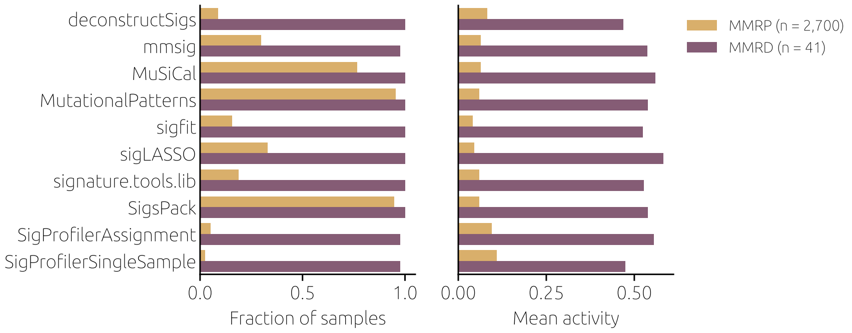


Supplementary Figure 25: Mutational signature analysis of PCAWG samples that have been clasiffied as mismatch repair proficient MMRP, $n = 2,700$) and deficitent (MMRD, $n = 41$) in https://doi.org/10.1038/s41588-024-01659-0 (see Supplementary Table 4). As in SF 24, we compute the fraction of MMRP and MMRD samples, respectively, where signatures associated with defective DNA mismatch repair (SBS6, SBS14, SBS15, SBS20, SBS21, SBS26, and SBS44) are active (left panel) and their mean joint activity in those samples (right panel).

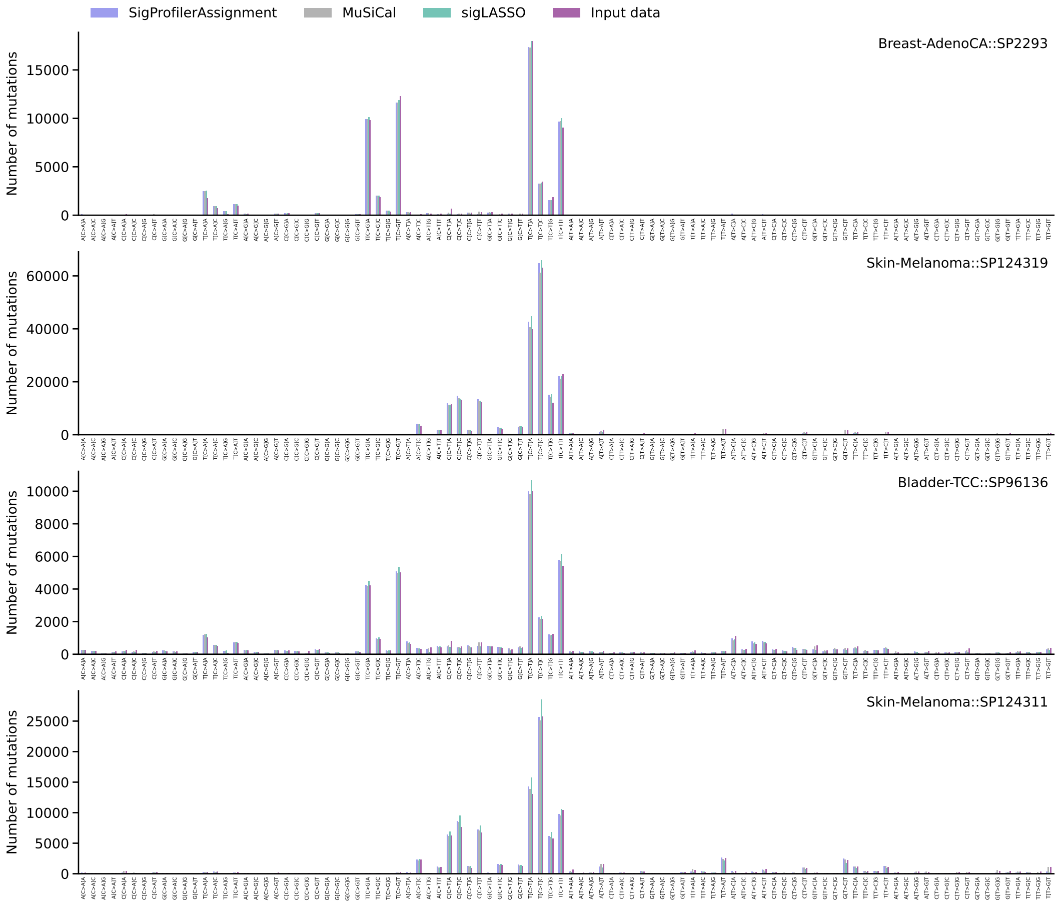


Supplementary Figure 26: Original and reconstructed mutational profiles of the four chosen real samples from the WGS PCAWG cohort.
