# Supplementary material for: A comprehensive comparison of tools for fitting mutational signatures
Source: Nat Commun. 2024 Nov 2;15:9467. doi: 10.1038/s41467-024-53711-6 (PMC11530434; doi:10.1038/s41467-024-53711-6)
Supplement: Supplementary file 2 — Reporting Summary [file 41467_2024_53711_MOESM2_ESM.pdf]

nature portfolio

Corresponding author(s): Matus Medo

Last updated by author(s): Sep 17, 2024

Reporting Summary

Nature Portfolio wishes to improve the reproducibility of the work that we publish. This form provides structure for consistency and transparency in reporting. For further information on Nature Portfolio policies, see our Editorial Policies and the Editorial Policy Checklist.

Statistics

For all statistical analyses, confirm that the following items are present in the figure legend, table legend, main text, or Methods section.

n/a	Confirmed	
☐	☒	The exact sample size (*n*) for each experimental group/condition, given as a discrete number and unit of measurement
☒	☐	A statement on whether measurements were taken from distinct samples or whether the same sample was measured repeatedly
☐	☒	The statistical test(s) used AND whether they are one- or two-sided<br>*Only common tests should be described solely by name; describe more complex techniques in the Methods section.*
☒	☐	A description of all covariates tested
☒	☐	A description of any assumptions or corrections, such as tests of normality and adjustment for multiple comparisons
☐	☒	A full description of the statistical parameters including central tendency (e.g. means) or other basic estimates (e.g. regression coefficient) AND variation (e.g. standard deviation) or associated estimates of uncertainty (e.g. confidence intervals)
☐	☒	For null hypothesis testing, the test statistic (e.g. *F*, *t*, *r*) with confidence intervals, effect sizes, degrees of freedom and *P* value noted<br>*Give P values as exact values whenever suitable.*
☒	☐	For Bayesian analysis, information on the choice of priors and Markov chain Monte Carlo settings
☒	☐	For hierarchical and complex designs, identification of the appropriate level for tests and full reporting of outcomes
☐	☒	Estimates of effect sizes (e.g. Cohen's *d*, Pearson's *r*), indicating how they were calculated

Our web collection on statistics for biologists contains articles on many of the points above.

Software and code

Policy information about availability of computer code

Data collection	No special software was used to collect data.
Data analysis	The code to generate synthetic mutational catalogs and evaluate signature fitting tools is available at https://github.com/8medom/SigFitTest. Links to the code for the evaluated fitting tools are provided in Table 2.

For manuscripts utilizing custom algorithms or software that are central to the research but not yet described in published literature, software must be made available to editors and reviewers. We strongly encourage code deposition in a community repository (e.g. GitHub). See the Nature Portfolio guidelines for submitting code & software for further information.

Data

Policy information about availability of data

All manuscripts must include a data availability statement. This statement should provide the following information, where applicable:

- Accession codes, unique identifiers, or web links for publicly available datasets
- A description of any restrictions on data availability
- For clinical datasets or third party data, please ensure that the statement adheres to our policy

The absolute contributions of signatures to sequenced tissues from various cancers are available from the Catalogue Of Somatic Mutations In Cancer (COSMIC), https://cancer.sanger.ac.uk/signatures/sbs/.

The reference catalogs of single base substitution (SBS) signatures are available from COSMIC, https://cancer.sanger.ac.uk/signatures/downloads/.

The real mutational catalogs are available from the International Cancer Genome Consortium (ICGC) upon request (the ICGC Data Portal where the data were previously publicly available has been recently closed down but the same data are available to all users who obtain access from the ICGC).

Research involving human participants, their data, or biological material

Policy information about studies with human participants or human data. See also policy information about sex, gender (identity/presentation), and sexual orientation and race, ethnicity and racism.

Reporting on sex and gender	Not relevant. We used real mutational catalogs (not full lists of mutations or sequencing data, just mutation classification using the SBS96 scheme) for some panels in Figure 4. These data were anonymized, obtained from a public ICGC repository, and we have not compared the samples based on their sex, race, or any other characteristics.
Reporting on race, ethnicity, or other socially relevant groupings	Not relevant. We used real mutational catalogs (not full lists of mutations or sequencing data, just mutation classification using the SBS96 scheme) for some panels in Figure 4. These data were anonymized, obtained from a public ICGC repository, and we have not compared the samples based on their sex, race, or any other characteristics.
Population characteristics	Not relevant. We used real mutational catalogs (not full lists of mutations or sequencing data, just mutation classification using the SBS96 scheme) for some panels in Figure 4. These data were anonymized, obtained from a public ICGC repository, and we have not compared the samples based on their sex, race, or any other characteristics.
Recruitment	Not relevant. We used real mutational catalogs (not full lists of mutations or sequencing data, just mutation classification using the SBS96 scheme) for some panels in Figure 4. These data were anonymized, obtained from a public ICGC repository, and we have not compared the samples based on their sex, race, or any other characteristics. The data were readily available, no recruitment took place.
Ethics oversight	Not relevant. We used real mutational catalogs (not full lists of mutations or sequencing data, just mutation classification using the SBS96 scheme) for some panels in Figure 4. These data were anonymized, obtained from a public ICGC repository, and we have not compared the samples based on their sex, race, or any other characteristics. The data were readily available, no recruitment took place.

Note that full information on the approval of the study protocol must also be provided in the manuscript.

Field-specific reporting

Please select the one below that is the best fit for your research. If you are not sure, read the appropriate sections before making your selection.

☒ Life sciences ☐ Behavioural & social sciences ☐ Ecological, evolutionary & environmental sciences

For a reference copy of the document with all sections, see nature.com/documents/nr-reporting-summary-flat.pdf

Life sciences study design

All studies must disclose on these points even when the disclosure is negative.

Sample size	We mostly used synthetic data in this work. The real data was used for Figure 4, panels a--c where the sample sizes are 4 (a and b) and 146 (c).
Data exclusions	From all available PCAWG catalogs, we used all catalogs with at least 50,000 mutations for panel c. For panels a and b, we use the four samples that have at least 50,000 mutations and three top-performing tools (SigProfilerAssignment, sigLASSO, and MuSiCal) agree on them. All these details are described in the manuscript.
Replication	We used a public ICGC repository to obtain the real mutational catalogs analyzed in panels 4a--c. We have not modified the data and our results can be replicated by anyone who will analyze the same dataset (available from the ICGC upon request) using the same tools. The SigFitTest package that we prepared includes the minimal data needed to replicate panels 4a and 4b.
Randomization	Not relevant (we have not used group allocation as groups of real samples were not compared).
Blinding	Not relevant (we have not used group allocation as groups of real samples were not compared).

Reporting for specific materials, systems and methods

We require information from authors about some types of materials, experimental systems and methods used in many studies. Here, indicate whether each material, system or method listed is relevant to your study. If you are not sure if a list item applies to your research, read the appropriate section before selecting a response.

Materials & experimental systems

n/a	Involved in the study
☒	☐ Antibodies
☒	☐ Eukaryotic cell lines
☒	☐ Palaeontology and archaeology
☒	☐ Animals and other organisms
☒	☐ Clinical data
☒	☐ Dual use research of concern
☒	☐ Plants

Methods

n/a	Involved in the study
☒	☐ ChIP-seq
☒	☐ Flow cytometry
☒	☐ MRI-based neuroimaging

Plants

Seed stocks	N/A
Novel plant genotypes	N/A
Authentication	N/A
